# Introduction of Syphilis Point-of-Care Tests, from Pilot Study to National Programme Implementation in Zambia: A Qualitative Study of Healthcare Workers’ Perspectives on Testing, Training and Quality Assurance

**DOI:** 10.1371/journal.pone.0127728

**Published:** 2015-06-01

**Authors:** Éimhín M. Ansbro, Michelle M. Gill, Joanna Reynolds, Katharine D. Shelley, Susan Strasser, Tabitha Sripipatana, Alexander Tshaka Ncube, Grace Tembo Mumba, Fern Terris-Prestholt, Rosanna W. Peeling, David Mabey

**Affiliations:** 1 Department of Clinical Research, London School of Hygiene and Tropical Medicine, London, United Kingdom; 2 Elizabeth Glaser Pediatric AIDS Foundation, Washington, District of Columbia, United States of America; 3 Department of Social & Environmental Health, London School of Hygiene and Tropical Medicine, London, United Kingdom; 4 Department of Epidemiology and Biostatistics, George Washington University, Washington, District of Columbia, United States of America; 5 Elizabeth Glaser Pediatric AIDS Foundation, Lusaka, Zambia; 6 Office of Population and Reproductive Health, United States Agency for International Development, Washington, District of Columbia, United States of America; 7 HIV/AIDS STI Programme, Ministry of Health, Lusaka, Zambia; 8 Department of Global Health and Development, London School of Hygiene and Tropical Medicine, London, United Kingdom; Johns Hopkins University, UNITED STATES

## Abstract

Syphilis affects 1.4 million pregnant women globally each year. Maternal syphilis causes congenital syphilis in over half of affected pregnancies, leading to early foetal loss, pregnancy complications, stillbirth and neonatal death. Syphilis is under-diagnosed in pregnant women. Point-of-care rapid syphilis tests (RST) allow for same-day treatment and address logistical barriers to testing encountered with standard Rapid Plasma Reagin testing. Recent literature emphasises successful introduction of new health technologies requires healthcare worker (HCW) acceptance, effective training, quality monitoring and robust health systems. Following a successful pilot, the Zambian Ministry of Health (MoH) adopted RST into policy, integrating them into prevention of mother-to-child transmission of HIV clinics in four underserved Zambian districts. We compare HCW experiences, including challenges encountered in scaling up from a highly supported NGO-led pilot to a large-scale MoH-led national programme. Questionnaires were administered through structured interviews of 16 HCWs in two pilot districts and 24 HCWs in two different rollout districts. Supplementary data were gathered via stakeholder interviews, clinic registers and supervisory visits. Using a conceptual framework adapted from health technology literature, we explored RST acceptance and usability. Quantitative data were analysed using descriptive statistics. Key themes in qualitative data were explored using template analysis. Overall, HCWs accepted RST as learnable, suitable, effective tools to improve antenatal services, which were usable in diverse clinical settings. Changes in training, supervision and quality monitoring models between pilot and rollout may have influenced rollout HCW acceptance and compromised testing quality. While quality monitoring was integrated into national policy and training, implementation was limited during rollout despite financial support and mentorship. We illustrate that new health technology pilot research can rapidly translate into policy change and scale-up. However, training, supervision and quality assurance models should be reviewed and strengthened as rollout of the Zambian RST programme continues.

## Background

12 million new cases of syphilis occur each year, the majority in developing countries [1]. Probable active syphilis occurs in 1.5 million pregnancies and contributes to 305,000 neonatal deaths and stillbirths each year [2,3]. Half of pregnant women with untreated syphilis will experience adverse pregnancy outcomes, such as miscarriage, stillbirth, premature delivery, low birth weight and neonatal infection [4–8]. Congenital syphilis has been a neglected public health problem in Sub-Saharan Africa (SSA), where syphilis prevalence among pregnant women ranges from 1.4 to 17% [9–12].

Universal syphilis screening of pregnant women is recommended as part of the basic antenatal care (ANC) package promoted by the World Health Organization (WHO), since the symptoms of early syphilis too often go unnoticed and late stages of the disease may be completely asymptomatic [13]. Despite the availability of screening tests and ample evidence that antenatal testing and treatment with single-dose Benzathine Penicillin improves pregnancy outcomes and is highly cost-effective, screening rates range from 1.7 to 79.9% of women attending antenatal care in SSA [6,14–21]. A recent analysis of antenatal surveillance data estimated 66% of syphilis-associated adverse pregnancy outcomes occurred in ANC attendees who were either not appropriately tested or treated for syphilis [3].

A number of key barriers to implementation of universal antenatal syphilis screening have been identified: inconsistent supply chain, patient cost and need to return for results, health worker absence or insufficient training, low prioritisation by health policy implementers and use of Rapid Plasma Reagin (RPR) as the standard diagnostic tool, which requires laboratory capacity, cold storage and electricity [6,11]. To address these barriers, new health technologies: rapid, specific and validated point-of-care (POC) syphilis tests, have been developed and successfully implemented in a variety of clinical settings [14,21–24].

However, experience gained in human immunodeficiency virus (HIV) and malaria treatment programmes illustrates that ensuring POC test use and reliability of results, particularly on scale-up, presents its own set of challenges [25–29]. Reliability is aided by adopting high quality test kits that are easy-to-use; providing adequate training to all healthcare workers (HCW); and integrating Quality Assurance/Quality Control (QA/QC) and supervision systems into programmes from the outset [23,26,30–32]. POC test implementation involves shifting testing to non-laboratory settings and non-laboratory HCWs, often unaccustomed to performing tests or routine QA/QC. Such task-shifting has important planning implications for workload of HCWs in already burdened health systems, and for testing strategy, diagnostic algorithms, QA/QC continuity and supply chain management [25,31,33–35]. Furthermore, HCW acceptance of POC tests and trust in their accuracy are key to ensuring that tests are performed and results are acted upon [25,27,36,37].

In this paper, we explore the end-user experience of new health technology introduction. We compare Zambian HCWs’ experience of RST usability, training and quality systems during a highly-supported pilot project versus a pared down national implementation programme. Through HCW and key informant interviews, we examine how health system planning and infrastructure influenced implementation of both pilot and first phase of Zambia’s national RST programme.

## Methodology

### Study design

#### Study context

Zambia is a lower-middle income country with a population of 14.1 million, spread over a large geographical area [38]. 94% of pregnant women attend at least one antenatal visit and ANC is free-of-charge [18,39]. The health care system is arranged in six tiers: outreach services, health posts, urban and rural health centres, district hospitals, secondary referral and tertiary referral hospitals [40]. Rural facilities are remote, located several hours’ drive from district hubs, lacking or with limited electricity and limited transport to convey patient samples, results or supplies back and forth to district laboratories.

In 2010–11, a six-country pilot study, including Zambia, evaluated the feasibility of introducing RSTs into existing maternal and child health (MCH) programmes [1,13,34]. The study was the first of its kind to incorporate QA systems to monitor training and accuracy of POC test results [34]. The non-governmental organisation (NGO)-led Zambian study arm introduced RST into existing prevention of mother-to-child transmission (PMTCT) of HIV services in 15 sites in two districts: Mongu, a rural district, with a syphilis prevalence of 7%, and Lusaka, an urban district, with a prevalence of 2.5% [41]. It showed that integrating RST into PMTCT programmes increased testing and treatment for syphilis in HIV positive pregnant women without compromising HIV service delivery.

Following the pilot’s success, the Zambian Ministry of Health (MoH) rapidly adopted RST into national policy and led the rollout of a national RST programme, incorporating QA/QC into programme design [42]. The rollout, supported by the Elizabeth Glaser Pediatric AIDS Foundation (EGPAF), commenced in 2012, initially in four underserved districts with high rates of maternal mortality: Kalomo, Lundazi, Mansa and Nyimba. Key implementation changes were made between pilot and rollout: supervision was reduced from monthly to quarterly and QA/QC activities were devolved from central to district laboratory level (Table 1).

**Table 1:** Changes in implementation methods from NGO-led RST pilot to MoH-led national RST rollout in Zambia 2008–2012.

	PILOT PHASE	ROLLOUT PHASE
Study Period	2008–2011	2012 to present
**Implementers/ Funders**	EGPAF, through external funders, and Center for Infectious Disease Research in Zambia implemented the Zambian arm of a six-country pilot coordinated by WHO /TDR Sexually Transmitted Diseases Diagnostic Initiative evaluating feasibility of RST introduction into prevention-of-mother-to-child-transmission of HIV (PMTCT) services.	EGPAF/CIDRZ collaborated with Zambian MoH technical working group to incorporate RST into national policy and produce national guidelines in 2011. EGPAF supported the MoH to implement first phase of national rollout by leveraging implementation funds and sharing pilot phase experience.
**Study Site**	15 facilities in 2 districts: Lusaka (urban; low syphilis prevalence: 2.5%); Mongu (rural; high syphilis prevalence: 7%)	All MoH facilities in 4 underserved districts with high rates of maternal mortality: Kalomo, Lundazi, Mansa, Nyimba.
**Training Model**	**Cascaded training:** Central workshop conducted by EGPAF/CIDRZ; attendees then provided on-the-job training to facility colleagues.	**Cascaded training:** District-level workshops conducted by MoH/EGPAF; attendees then provided on-the-job training to facility colleagues.
**Treatment algorithm**	Zambian syphilis treatment guideline pre-RST adoption was three weekly doses of Benzathine Penicillin (BP). During the pilot, patients were given one documented dose of BP following a positive RST test.	Treat with one dose of BP following positive RST result; run RPR confirmation and if active infection confirmed, treat with two additional doses of BP. If RPR confirmation unavailable, continue 2^nd^ and 3rd weekly dose of BP.
**Integration into existing services**	RST integrated into existing staffing patterns and patient flow; alongside other routine antenatal tests (HIV, malaria, and haemoglobin).	RST integrated into existing staffing patterns and patient flow; alongside other routine antenatal tests. Rollout included MoH, Ministry of Defense and mission-affiliated facilities.
**Outcome of integration**	The same HCW offered same-day testing, results and treatment.	RST was variably integrated into patient flow depending on facility-level, HCW cadre and laboratory capacity.
**Quality Control**	**Internal QC**: in-built control panel **External QC:**1)weekly validation of RST kits with positive and negative control samples; 2) repeat confirmatory testing at a central laboratory of 2% of samples collected during study supervision visits.	**Internal QC**: in-built control panel **External QC:** Validation of RST kits with positive and negative control samples weekly and if a new shipment, new lot number or adverse environmental conditions occurred. Rarely implemented during early rollout phase evaluated here. Knowledge on QA/QC practices was rarely transferred during cascaded training. Control samples were not included in test kits or delivered to facilities by district lab personnel.
**Quality Assurance**	Health workers’ accuracy was checked using **proficiency panels** (sample RSTs prepared with dried tube specimens of serum known to be positive or negative for syphilis) during supervisory visits	Health workers’ accuracy was intended to be checked using **proficiency panels** sent to the facility by the district laboratory. Not implemented during the early rollout phase evaluated here due to lack of HCW time, lack of dedicated budget, logistics and manpower for QA/QC, inexperience and/or lack of initiative of the district laboratory personnel and lack of on-site lab-training in advance of rollout.
**QA/QC Logistics**	Control samples prepared by research laboratory and transported by study staff to facilities	Control samples were intended to be prepared by district laboratories and transported to the facilities with results transported back to the district laboratory. Not implemented during the early rollout phase examined in this study.
**Supervision**	**Monthly** visits from EGPAF study staff incorporating QA and remedial training for new or poorly performing HCWs	**Quarterly** visits from MoH and EGPAF staff
**Cost Implications**	QA/QC activities contributed significantly to pilot costs, driven by central-level personnel supervision and transport costs	QA/QC rollout costs were reduced due to decentralisation of supervision and quality monitoring to the district level; costs were driven up by higher RST kit cost during rollout and reduced economies of scale due to reduced RST uptake.

**Fig 1:**
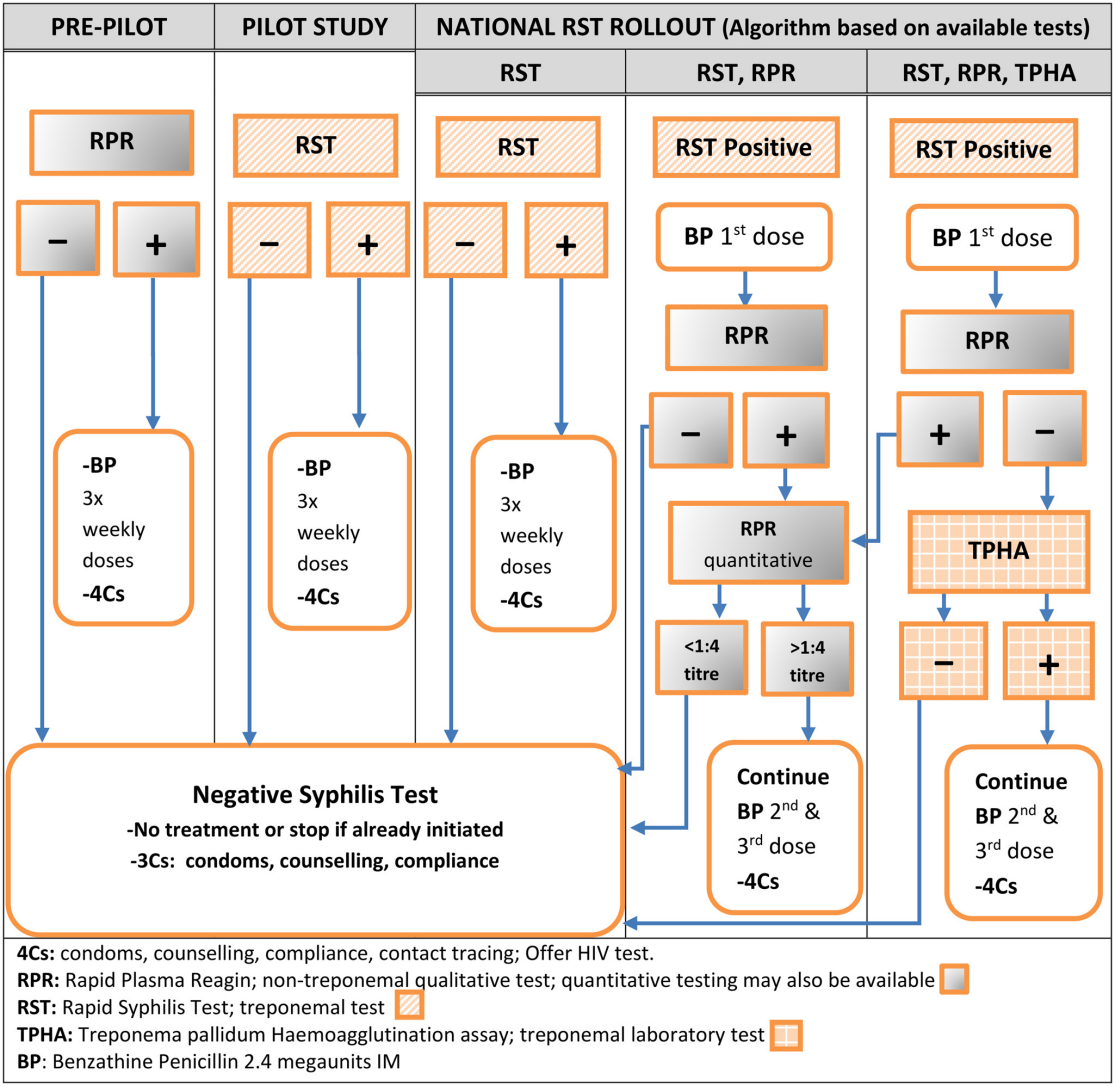
Phased changes in syphilis testing and treatment algorithm in Zambia 2009–2012. Legend: 4Cs: condoms, counselling, compliance, contact tracing, offer HIV test; RPR:Rapid Plasma Reagin; non-treponemal qualitative test; quantitative testing may also be available;RST: Rapid Syphilis Test, a treponemal test;TPHA*:Treponema pallidum Haemoagglutination assay, a treponemal laboratory test;BP: Benzathine Penicillin 2.4 megaunits IM. *The testing algorithm involving TPHA applied to tertiary care centres and was unavailable at sites included in this evaluation.

Prior to the pilot, antenatal syphilis screening was carried out using the laboratory-based RPR test when available. MCH HCWs sent blood samples to a laboratory for batched testing, with patients usually returning for results at a later date. In some rural facilities, non-laboratory workers performed RPR tests without a systematic QA approach. During the pilot, RST was performed by MCH HCWs who started same-day treatment based on a reactive RST test. National rollout guidelines described several diagnostic algorithms, depending on RST, RPR and Treponema pallidum haemoagglutination (TPHA) test availability at each site (Fig 1). None of the sites included in this evaluation performed TPHA.

#### Data collection

This qualitative evaluation used a HCW questionnaire (S1 Appendix) and key informant interviews to determine the feasibility and acceptability of introducing RST into PMTCT services, comparing pilot and national rollout implementation phases. The concept of “feasibility”, drawn from the health technology literature, was defined as the process of RST programme implementation leading to end-user acceptance and utilisation, discussed further below [37]. Acceptability was defined as health workers’ positive satisfaction levels and their correct and consistent use of RST [41]. A health economic analysis of pilot and rollout phases is presented in our accompanying paper, Shelley et al. [43].

The questionnaire utilised both closed questions (including five-point Likert scales) and open-ended questions eliciting qualitative responses. The following domains were included: RST advantages and disadvantages, patient experience, organisational environment and workflow, training, skill and confidence in test performance and RST acceptability. For the rollout evaluation, the pilot questionnaire was adapted using current literature on technology acceptance; domains on QA/QC and supervision were added; and a topic guide was designed, covering the same domains as the questionnaire. Further adjustment, following a pilot interview and feedback from completed MoH/EGPAF supervisory visits, strengthened the internal validity of the study.

During the pilot assessment, SAS software (version 9.1) was used to randomly select four sites, two in each study district. They included a district hospital and both low and high volume health centres. Data on service and patient volumes, staffing numbers and location, time/motion studies and cost data were collected to document changes in syphilis and HIV testing and treatment and are reported elsewhere [41]. For the rollout evaluation health facilities were selected by convenience sampling (based on MoH guidance and practical limitations such as distance, staffing and vehicle availability) to reflect a range of urban and rural clinical settings with varying laboratory capacity. Review of records and key informant interviews took place at District Health Offices (DHO).

Face-to-face interviews were conducted in English with consenting health workers, with the exception of one interview, which used a translator. In November and December 2010, two EGPAF study staff administered the questionnaire to pilot HCWs, including seven midwives, two nurses and seven lay counsellors. Twenty four rollout HCWs (including four midwives, four nurses, five lay counsellors, one clinical officer, six laboratory technicians, three environmental health technologists and one psychosocial counsellor) were interviewed in August 2012 by one of the authors (É.A.), an independent researcher who accompanied MoH/EGPAF on supervisory visits. Responses were hand-written and rollout interviews were also audio-recorded and subsequently transcribed by the interviewer. Interviewees were selected by convenience sampling, aiming to interview four HCWs at each pilot site and any HCW who had ever performed RST at each rollout site. Using the MoH guideline, any errors identified in test performance were immediately remediated post-interview and discussed with the implementing partner. Informal interviews were held with key informants during MoH supervisory visits to gain understanding of the planning, implementation and costs of the national RST programme. Key informants included EGPAF senior and pilot study staff, MoH HIV/AIDS STI Programme staff, and DHO staff from Mansa and Kalomo districts. Data were collected in the form of field notes recorded after each informal interview.

#### Conceptual framework and analysis

**Fig 2:**
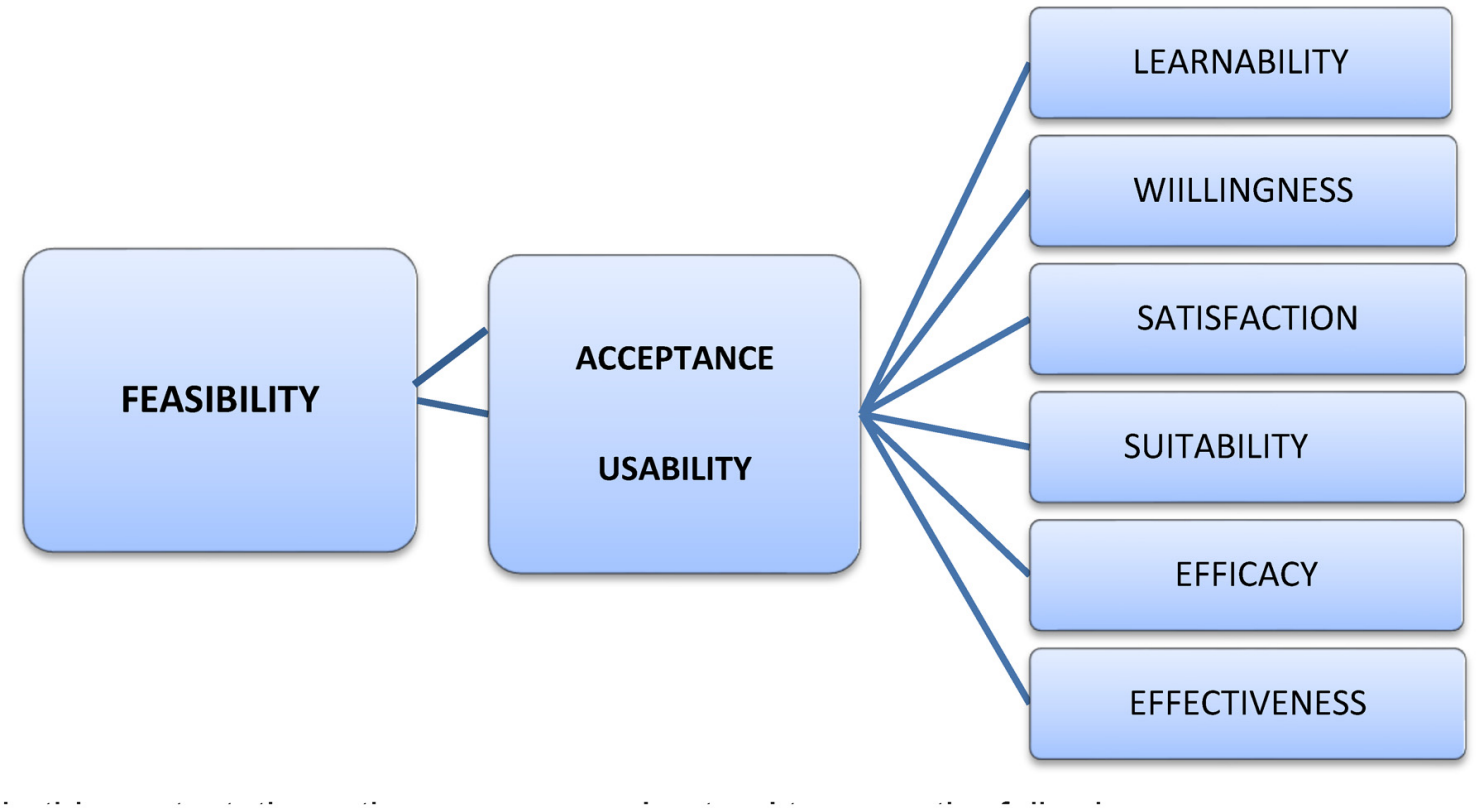
Conceptual framework for the evaluation of RST pilot study and national programme in Zambia, adapted from Asiimwe et al (2012). Legend: In this context, these themes were understood to mean the following: Learnability: how easy or difficult it was for HCW to learn to perform the RST, perform it accurately and learn about quality control and quality assurance; Willingness: Willingness of the HCW to perform the RST, to take part in the cascaded training i.e. being trained by or training other colleagues; willingness to take part in supervisory and quality assurance activities; Suitability: HCWs’ belief the RST test was relevant to their work and could be successfully integrated into existing services; HCWs’ belief in the appropriateness of the current supporting components of the RST programme i.e. training, supervision and quality maintenance; Satisfaction: HCWs’ satisfaction with the test itself, its impact on workflow and satisfaction with the supporting components of the programme; Efficacy: Ability of HCWs to implement same-visit testing and treatment (STAT), to incorporate the test and to integrate quality assurance and quality control activities into their workflow; Effectiveness: How the organisational and systemic environment, including implementation of policy, guidelines, supply chain and other logistics, impacted on successful delivery of the programme. In addition, how the social context (the community, patients and their partners) influenced programme delivery.

A conceptual framework was created to guide the analysis, based on a model used by Asiimwe et al. which explored the feasibility of new health technology introduction. The framework divides the concept of feasibility into two inter-related domains, *acceptance* and *usability* [37]. Technology acceptance and usability originate in the study of human-computer interaction. Usability has been further broken down into various attributes [44,45]. Here, acceptance and usability were divided into six sub-domains: *learnability*, *willingness*, *suitability*, *satisfaction*, *efficacy* and *effectiveness*, attributes which have been described in other settings (Fig 2) [45]. The framework recognises that acceptance and usability may be influenced by factors related to the end-user (both HCW and client), the diagnostic tool and the health system. Health system factors include guidelines and training, quality monitoring and evaluation, supply chain, and policy, budget and planning.

Data from the two evaluation phases were collected, entered and analysed by separate pilot and rollout teams. Quantitative data were double-entered, verified and cleaned using MS Access (2007–10) and Microsoft Excel 2003 respectively (S1 Dataset). Qualitative data were entered into MAXqda (V10) and NVivo 10, respectively, which are computer-assisted qualitative data analysis software packages. Two authors (M.G. and É.A.) used template analysis to independently analyse qualitative data from each phase [46]. The initial coding template was based on the questionnaire and included codes for: HCW acceptance and satisfaction, patient and partner experience of RST, training, workflow and integration with existing services and quality assurance activities and supervision. For this paper, the authors compared data from each phase under these theme headings and explored and refined emerging sub-themes through iterative discussion.

### Ethical considerations

The pilot study protocol and consent procedures were approved by the University of Zambia Biomedical Research Ethics Committee (UNZAREC), the University of Alabama at Birmingham’s Institutional Review Board, the World Health Organization Research Ethics Review Committee and the Zambian MoH. The rollout study protocol and consent procedures were approved by UNZAREC, the London School of Hygiene and Tropical Medicine Research Ethics Committee and the Zambian MoH. To minimise use of personal identifiers, verbal informed consent was obtained from pilot HCWs and recorded on an approved consent form by interviewers. During rollout evaluation, written informed consent was obtained from each relevant District Health Officer and from each interviewed HCW using an approved consent form; verbal informed consent to participate was obtained from key stakeholders and recorded by the interviewer.

## Results

In evaluating the pilot, 16 HCW interviews were conducted at four sites, located in two study districts (Mongu and Lusaka). In the rollout evaluation, 24 HCW interviews were conducted in two facilities in Kalomo and five facilities in Mansa (Table 2). Informal interviews were conducted with four senior administrative and management staff in each DHO. Using the conceptual framework, we compare and contrast results from pilot study and rollout evaluation according to the following themes: RST acceptance and satisfaction; RST usability; training and guidelines; quality assurance activities and supervision; health system and supply chain.

**Table 2:** Health care worker and facility data for pilot and rollout evaluation phases.

	PILOT: March—July 2010				ROLLOUT: March—July 2012								
Facility Type^	UHC5	UHC6	RHC6	RHC7	UCH7	DH1	RHC3	RHC4	RHC6	UHC4	UHC8	RHC8	RHC9
Province	Lus	Lus	Wes	Wes	Sou	Sou	Lua	Lua	Lua	Lua	Lua	Lua	Lua
District	Lus	Lus	Mon	Mon	Kal	Kal	Man	Man	Man	Man	Man	Man	Man
Total Facility ANC* workers	n/a	n/a	n/a	n/a	10	41	18	4	2	n/a	n/a	n/a	18
HCW workshop-trained	1	1	1	1	1	6	1	1	1	3	1	1	1
HCW trained on-the-job	n/a	n/a	n/a	n/a	0	5	4	2	1	7	2	3	3
% total ANC staff trained	n/a	n/a	n/a	n/a	10	27	28	75	100	10	3	4	22
1st ANC Clients	n/a	n/a	n/a	n/a	n/a	638	271	144	n/a	480	n/a	n/a	n/a
Women screened with RST	n/a	n/a	n/a	n/a	n/a	154	103	114	n/a	430	n/a	n/a	n/a
% 1st ANC Clients screened	n/a	n/a	n/a	n/a	n/a	24.1	38	79.2	n/a	89.6	n/a	n/a	n/a
% of tests reactive	n/a	n/a	n/a	n/a	n/a	7.1	3.9	3.5	n/a	56.3	n/a	n/a	n/a
Number HCW interviewed	4	4	4	4	3	4	2	2	1	5	3	3	1
Cadre of HCW interviewed	4 MW	2 MW	MW	Nu	CO	Nu	EHT	Nu	EHT	3 MW	3 LT	Nu	PC
		2 LC	2 LC	3 LC	Nu	MW	LC	LC		LT		EHT	
			Nu		LC	2 LT				LC		LC	
Laboratory capacity?	yes	yes	no	no	no	yes	yes	no	no	yes	yes	no	no
RST kits available on interview day?	yes	yes	yes	yes	yes	yes	no	no	no	yes	no	yes	yes
RPR ever performed at facility?	yes	yes	yes	no	yes	yes	yes	yes	no	yes	yes	no	no

*ANC (Antenatal Care) worker includes all of the cadres listed below.

HCW: healthcare worker; CO: clinical officer; LC: lay counsellor; Nu: nurse; LT: laboratory technician/microscopist; MW: midwife; PC: psychosocial counsellor; RST: rapid syphilis test; RPR: rapid plasma reagin test; UHC: urban health centre; RHC: rural health centre; DH: district hospital; Lus: Lusaka; Wes: Western; Sou: Southern; Lua: Luapula; Mon: Mongu; Kal: Kalomo; Man: Mansa.

^^^See accompanying paper, Shelley et al., for additional facilities where only costing evaluation was performed.

### RST acceptance and satisfaction

Overall, both pilot and rollout HCWs *accepted* RST as a *suitable* addition to existing services. One rollout clinical officer stated:

*“I would encourage the MoH to embrace it and to roll it out in the whole country*.*”*

Most HCWs were strongly motivated by the opportunity to deliver a better service and offer *effective*, same-day testing and treatment (STAT) to patients to ensure safer pregnancies. They reported perceived increases in testing rates and treatment access for pregnant women and their partners as well as perceived reductions in loss to follow-up and adverse pregnancy outcomes. They were *satisfied* with RST reliability and accuracy.

HCWs in both phases reported patient *satisfaction* with the RST but this was notably less in the rollout: 93.8% of pilot HCWs (15/16) and 62.5% of rollout HCWs (15/24) thought patients were somewhat or very accepting of the RST. A rollout lay counsellor commented that patients: “*received (it) with both their hands*.” Patient benefits reported by HCWs in both phases included: STAT, reduced clinic waiting time, reduced travel time and increased case detection and treatment. Reduced patient waiting time was more evident in the pilot; 68.8% (11/16) of pilot HCWs reported a reduction compared to 39.1% (9/23) of rollout HCWs. By contrast, several (26%, 6/23) rollout HCWs reported an increase in patient waiting times following RST introduction.

POC technology allows patients to directly observe their test result. Rollout HCWs reported that this, along with patient counselling, assisted in alleviating patients’ initial distrust of RST:

*“Every change is quite hard to comprehend…We have to try hard so…people really adapt to new situations*,*”*

rollout lay counsellor.

HCWs encountered some difficulties communicating RST test results to patients, in cases where tests were positive due to previously treated infection (and, therefore, requiring only a single dose of BP) or in cases of partner sero-discordance. Counselling was referred to repeatedly by all rollout interviewees as key to client and partner acceptance of testing and treatment. Many drew on their HIV counselling training.

### RST usability

#### Health technology usability

Both pilot and rollout HCWs found the RST highly learnable; prior experience with POC tests added to their confidence. They rated the individual steps in test performance as easy or very easy.

*“The [RST] procedure is simple*, *good and very short*,*”*

pilot lay counsellor.

Compared to RPR testing, it was less “cumbersome” and time-consuming to perform, and easier to interpret. The mobility and convenience of RST allowed each stage of syphilis testing and treatment to be performed in one facility department, or out in the community:

*“It’s like a supermarket*, *we do everything here…HIV test*, *results…Hb*, *the syphilis… the same day*,*”*

rollout midwife.

However, some aspects of RST were less user-friendly. HCWs in both phases described difficulties reporting results within the appropriate time-frame and with interpreting results. These difficulties were more pronounced during rollout: only 30% (7/23) of rollout HCWs rated reporting results within the correct time-frame as “very easy” compared to 87.5% (14/16) of pilot HCWs; half of rollout HCWs were unsure how to correctly interpret weak positive results, which should be reported as positive. Several described repeating the RST or referring to the laboratory for confirmation of weak positive results; neither practice is in accordance with guidelines.

#### Usability—integration within existing services

Before RSTs were introduced at study sites, HCWs reported that, despite inclusion in national ANC guidelines, antenatal syphilis testing using RPR was inconsistently performed. RPR was performed at only 3 of 4 pilot sites and 6 of 9 rollout sites during the interviews. Most HCWs agreed RSTs were successfully integrated into facility PMTCT services, (16/16 pilot and 23/24 rollout HCWs) but there was high inter-site variability in algorithm followed and effect on workflow. The pilot integration pattern was standard: a MCH HCW (midwife, nurse or lay counsellor) ran both RST and HIV tests and returned results; a midwife or nurse then initiated treatment thus *efficiently and effectively* providing STAT. RPR confirmation was not included in the pilot protocol.

By contrast, integration at rollout sites depended on the facility’s health system level, available HCW cadres and laboratory capacity. Fewer rollout HCWs reported consistent delivery of STAT (83%, 20/23) compared with pilot HCWs (100%, 16/16). This may in part be due to variable patterns of task shifting. At larger facilities HCWs reported splitting testing steps amongst different HCWs and different departments, which led to delays in returning results and initiating treatment. In some cases confirmatory RPR was performed by the same HCW; in others, clients were required to return for RPR testing or results. Notably, midwives in one rollout facility reported a high reactivity rate of 56.3% on initial RST introduction. To verify results, they repeated RST or referred to the laboratory, where the lab technician reported repeating RST followed by RPR on all positive ANC samples, out of keeping with guideline algorithms. It also proved initially confusing to some rollout HCWs that the treatment algorithm differed in the case of negative RPR (Fig 1).

Pilot HCWs regarded RST as a user-friendly addition to PMTCT services which resulted in overall time-savings, despite the additional time required to counsel patients on both syphilis and HIV:

“*…results are out in a short time…thus*, *cuts on time we spend on syphilis testing giving us more time to do other routine activities in MCH*,*”*

pilot lay counsellor.

**Fig 3:**
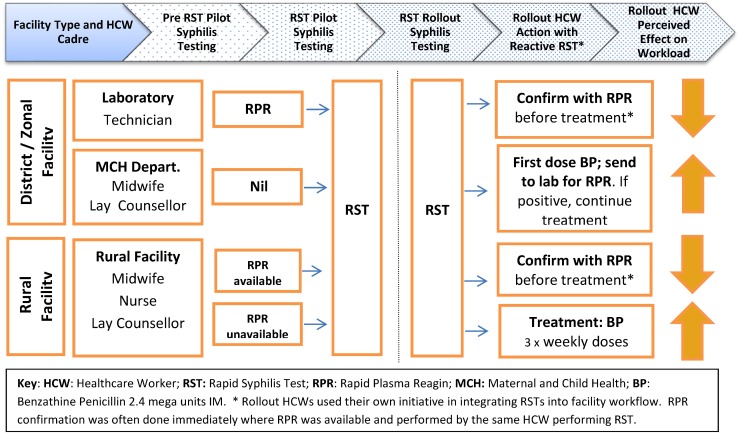
Changes in Syphilis Testing before and after RST introduction and perceived effect on workload for different HCW cadres in Zambia. **HCW**: Healthcare Worker; **RST:** Rapid Syphilis Test; **RPR**: Rapid Plasma Reagin; **MCH:** Maternal and Child Health; **BP**: Benzathine Penicillin 2.4 mega units IM. *Rollout HCWs used their own initiative in integrating RSTs into facility workflow. RPR confirmation was often done immediately where RPR was available and performed by the same HCW performing RST.

In the rollout, the effect on workload depended on both the role the HCW had in syphilis testing prior to RST introduction and the algorithm employed (Fig 3). Those previously involved in RPR testing reported overall time-savings irrespective of whether they then went on to perform RPR for some clients; those *not* previously involved felt RST introduction increased their workload and staffing levels were insufficient to manage:

*“I have a lot of clients …I have to see them… counsel them and do the RST to the woman and the man*. *So*, *it becomes difficult at times when I’m alone*. *That is in terms of staffing*,*”*

rollout midwife.

As a result, they deferred syphilis testing to a later ANC visit or delayed reading test results.

### Training and guidelines

Training experiences varied between pilot and rollout, with pilot HCWs more likely to have received training from EGPAF staff. 25% (4/16) of pilot interviewees reported attending an EGPAF training workshop; the remainder received on-the-job training from colleagues or EGPAF study staff; half received remedial booster training from EGPAF during monthly supervisory visits. 29% (7/24) of rollout interviewees attended the MoH/EGPAF-led training workshop. The remainder (17/24) received on-the-job training from colleagues.

According to MoH staff, the rollout training workshop emphasised on-the-job training but did not include specific “train-the-trainer” techniques. A training manual was provided to workshop attendees but few reported using it to train colleagues. Following training, only three (3/23) rollout HCWs used SOPs to aid accurate testing; all (16/16) pilot HCWs used them.

Most rollout HCWs found both workshop and on-the-job training acceptable. Several HCWs believed on-the-job training was too short and contained inaccurate or incomplete “second-hand information”. Some HCWs described colleagues’ distrust of them as trainers, their *dissatisfaction* at missing “official” training and their resultant *unwillingness* to perform the test:

*“*...*they think*, *since you’re the one who went to the workshop you will be the one to do (the RST)*,*”*

rollout counsellor.

### Quality assurance activities and supervision

*Acceptance* and *usability* of QA/QC activities were investigated as part of the rollout evaluation only. 100% of rollout respondents *accepted* QA/QC activities and recognised the potential benefits:

*“I think it will help us know if the test kits are working properly and you would be confident that the result you would give would be the correct result*,*”*

rollout midwife.

However, few rollout HCWs accurately recalled the QA/QC programme components described in RST training manuals. A small number reported performing internal QC to check test kit quality (e.g. ensuring the test control window was positive before reporting results). With the exception of one PMTCT counsellor who displayed self-*efficacy* in using RPR control samples to check kits, no HCW reported performing systematic external QC or completing proficiency panels.

Guidelines stated that district laboratories should prepare and provide positive and negative control samples for external QC and dried-tube specimens for proficiency panel testing. Laboratory facilities reported performing their own weekly QC; one district laboratory sent proficiency panels to local facilities but no results were returned. Reported barriers to the provision of district-led QA/QC were lack of trained staff, dedicated time, transport and reporting infrastructure.

Supervision differed from pilot to rollout. EGPAF made monthly support visits during the pilot and supported the MoH on quarterly supervisory visits during rollout. Rollout HCWs expressed *willingness* to undergo supervisory visits, describing them as an opportunity to learn and to correct mistakes. Most believed supervision was a *suitable* and *effective* tool, which boosted morale and encouraged HCWs to work more diligently and more accurately. Some *dissatisfaction* with supervision was expressed; a minority felt demoralized, intimidated or uncomfortable when observed performing tests.

### Health system and supply chain

Supply of penicillin was consistent during the pilot but was less reliable during rollout: 12.5% (3/24) of rollout interviewees said stock-outs occurred once a month. RST kit supply was also less reliable during rollout; half of pilot sites (2/4) reported a stock-out on one occasion whereas almost a third of rollout sites (7/24) reported a complete RST stock-out during the month preceding interviews. EGPAF key informants reported that RST kits were not supplied through the usual national supply chain but rather through the DHO Pharmacy. This was to avoid incorrectly delivering to districts not yet involved in rollout but it led to confusion and ruptures in RST supply.

## Discussion

This two-phased evaluation provides a detailed account of new POC test implementation, examining the transition from an NGO-led pilot project to the first phase of a national RST programme in Zambia. Novel, inexpensive and easy-to-use POC tests can address inequality in access to diagnostics in lower and middle income countries such as Zambia. However, implementation can be complex and requires a usable test, acceptance by HCWs and patients, good quality training, ongoing HCW support, quality monitoring systems and robust supply chains, which are more difficult to ensure for large-scale rollout with budget constraints [25,33,37,47]. Jafari et al described a lack of quality reporting of implementation research outcomes, other than accuracy, for syphilis POC tests, which our study helps to address [48]. This early programme evaluation takes place at multiple health system levels in four Zambian districts and may inform the Zambian MoH as they plan further phases of RST programme rollout.

Using HCW surveys and key stakeholder interviews, we examined the success of RST implementation from the perspective of “feasibility”, a concept borrowed from the health technology literature, which was further broken down into two components: “acceptance” and “usability” [37,45]. This study was not designed to report impact of RST introduction into PMTCT services in Zambia, which is reported elsewhere [41].

Overall, the study findings were positive with both pilot and rollout HCWs reporting that RST largely met our criteria for *acceptability* and *usability*. However, feasibility, as defined here, is contingent not only on end-user and technology attributes but also on health system and programmatic factors. We identified that several aspects of the rollout programme differed from the pilot, particularly training, testing algorithm, QA/QC, supervision, and supply chain; these may have negatively influenced the end-user experience and therefore the feasibility of the RST rollout programme.

Both groups found some aspects of RST non-user-friendly but rollout HCWs demonstrated significantly less correct and consistent use of RST, likely influenced by less exposure to MoH/NGO-provided training and regular supervision. Rollout on-the-job training quality appeared highly variable; the *effectiveness* of the cascaded training model utilised in the rollout was clearly limited. The inconsistent implementation of rollout testing and treatment guidelines may reflect the nature of the training and scaled-down supervision received by rollout HCWs compared to pilot HCWs and may have influenced the quality of care delivered. The POC literature emphasises the importance of high quality training to ensure test accuracy and reliability [25,30,31,49]. This is increasingly critical as HCWs are now expected to perform multiple rapid tests in parallel, each with varying specifications and manufacturers’ instructions. The WHO and global health workforce alliance advocate the cascaded model of training, or “train-the-trainer” (TTT) approach, as one way to meet the global health workforce crisis [50–52]. The effectiveness of TTT is supported by a recent systematic review. However, there is dearth of knowledge on TTT programme content, the training techniques that should be incorporated, or on programme sustainability [53].

Rollout HCWs were *willing* to take part in cascaded training, although some expressed resentment at missing the opportunity to attend an off-site training workshop. This may be linked to the widespread use of per diem payments for health workers in some low and middle-income countries, such that they have become an expected supplement, sometimes even exceeding health sector salaries. While these payments may encourage productivity and motivation, they have been shown to create conflict between HCWs and even to demotivate those who don’t receive them [54,55]. Supervision and monitoring of HCWs’ performance has been shown to increase their confidence in test performance and accuracy of test results [56], a finding echoed in this study. During the pilot, rates of both HIV and syphilis testing and treatment improved, which was attributed in part to regular supervision [34].

Overall, rollout HCWs were less *satisfied* with the increased workload resulting from the addition of another POC test to MCH services, but this was dependent on their previous role in syphilis testing and the algorithm employed. Our findings support a New Zealand study which showed that POC test introduction can have important, unforeseen implications for workforce planning [57]. Most rollout HCWs inaccurately described the steps involved in RST performance, confusing them with other POC tests. The assumption that integrating multiple testing programmes serves to strengthen health systems may not always hold true [34,58]. The new dual RST and HIV POC tests, which are currently under evaluation by the WHO and partners, may eliminate some of the problems engendered by running multiple POC tests in parallel [59]. Less frequent supervision may be one reason rollout HCWs reported greater difficulty with test performance and employed a variety of integration patterns, resulting in lower rates of STAT.

Rollout HCWs perceived lower levels of patient satisfaction than their pilot colleagues, which may result from the longer patient waiting times reported by some. HCWs also described difficulties around negotiating patient expectations and understanding of test results. Similar issues have been described in the literature on malaria rapid diagnostic test (RDT) introduction, particularly challenging patient expectations of malaria treatment in the face of a negative malaria test [28,29,36,60]. Specific training in counselling patients and partners around RST results may prove useful to include in further editions of RST training manuals.

The importance of QA/QC systems has been repeatedly emphasised in recent literature on HIV and malaria POC tests. Despite this, most HIV testing programmes in developing countries operate without them [61]. Ensuring reliable POC test results is of paramount importance as inaccuracies lead to incorrect diagnosis and treatment and negatively impact patient care and cost-effectiveness. Assuming the same issues regarding POC accuracy might beset RST implementation, QA/QC were incorporated into both pilot study and national rollout [26,49].

Anticipating that QA/QC could be an area of weakness during rollout, several modules of the MoH training guide were devoted to QA, QC and monitoring procedures [42]. All rollout HCWs interviewed understood the rationale for QA and demonstrated *willingness* to take part. However, the first round of rollout supervisory visits revealed limited implementation of these procedures. Several factors may have contributed to this: ineffective planning and communication (in that no specific person at facility or district level was nominated to coordinate QA/QC), lack of dedicated budget and logistics, lack of local expertise in this type of activity which is often undertaken by NGOs, or lack of *efficacy* on the part of local laboratory staff. This suggests that HCW training on QA/QC was not *learnable* or *effective* in its current form. Further research is needed to establish the ideal mix of RST QA/QC activities which is usable, acceptable, effective and cost-effective.

Supply chain issues may have impacted rollout success. For POC tests to maximise their potential, supplies of test kits, treatment and other consumables must be consistently available [25]. Weaknesses in both MoH district pharmacy and central medical supply chains were identified, which should be addressed before commencing further phases of the rollout.

Limitations of this study include the fact that it was a single-country case study whose findings may not be generalizable to other countries. Interviews and data analysis were performed by two separate research teams using slightly different collection tools which may reduce comparability of data from pilot and rollout phases. The pilot surveys were conducted by the implementing organisation and surveys in both phases were carried out during supervisory visits, increasing the risk of social desirability bias. The majority of study findings are based on a HCW survey; data are therefore response-based and susceptible to social desirability bias and measurement error. Convenience sampling of interviewees may have introduced selection bias. Quantitative data are also subject to measurement error. In addition this study design was not able to ascertain programme impact.

The Zambian RST scale-up model incorporated many success factors defined by Yamey in a recently proposed framework for successful scale-up of global health interventions [62]. However, we suggest that there are a number of aspects which ought to be refined before further rollout of the Zambian RST programme; our findings have been shared with the MoH and their implementing partner. The ideal training model, supervision type and frequency and mix of QA/QC activities for RST programmes has not been established and further implementation studies, which also examine impact, are required.

To strengthen the cascade model of training, the MoH could adopt a comprehensive “training of trainers” model incorporating adult education techniques and certifying workshop attendees as “local trainers”. Integrating training with HIV, malaria and Haemoglobin POC test training would facilitate emphasis on differing test characteristics and reduce HCW confusion. QA programme aspects would be strengthened by a distinct laboratory QA rollout strategy identifying specific responsible local personnel, and facilitating them with dedicated financing and logistics. Collaboration with those organisations already performing monitoring and evaluation of HIV testing may prove effective. Advocacy with the RST kit manufacturer to include positive and negative control samples could facilitate QC testing and address some of the difficulties experienced in communication and transport between laboratories and the surrounding health facilities. In addition, the supply chain must be strengthened.

## Conclusion

This study provides insight into the challenges of integrating new health technology into existing health systems and explores the transition from pilot study to national rollout. It illustrates programmatic experience of implementing cascaded training and QA, QC and supervision activities. Use of a conceptual framework adopted from a paper on health technology “usability” facilitated the exploration of programme implementation and may prove useful in other settings. We demonstrate that it is feasible to implement a MoH-led rapid syphilis testing programme in Zambia, following a successful pilot, but that issues around training, QA/QC and supply must be addressed in planning for future phases of this and other programmes incorporating POC tests. The Zambian MoH has already demonstrated commitment to monitoring and evaluation practices and addressing these programme aspects should serve to strengthen the health system as a whole.

## Supporting Information

S1 Appendix Topic Guide and Healthcare Worker Questionnaire. (PDF)

S1 Dataset Rollout Quantitative Dataset. (XLSX)

## References

[1] Department of Reproductive Health and Research, World Health Organization. WHO | The global elimination of congenital syphilis: rationale and strategy for action. In: WHO [Internet]. 2007 Available: http://www.who.int/reproductivehealth/publications/rtis/9789241595858/en/index.html

[2] World Health Organisation. Investment case for eliminating mother-to-child transmission of syphilis: promoting better maternal and child health and stronger health systems. In: WHO Press, World Health Organization, 20 Avenue Appia, 1211 Geneva 27, Switzerland [Internet]. 2012 [cited 15 Jan 2015]. Available: http://apps.who.int/iris/bitstream/10665/75480/1/9789241504348_eng.pdf

[3] Newman L, Kamb M, Hawkes S, Gomez G, Say L, Seuc A, et al Global estimates of syphilis in pregnancy and associated adverse outcomes: analysis of multinational antenatal surveillance data. PLoS Med. 2013;10: e1001396 10.1371/journal.pmed.1001396 23468598 PMC3582608

[4] Gomez GB, Kamb ML, Newman LM, Mark J, Broutet N, Hawkes SJ. Untreated maternal syphilis and adverse outcomes of pregnancy: a systematic review and meta-analysis. Bull World Health Organ. 2013;91: 217–26. 10.2471/BLT.12.107623 23476094 PMC3590617

[5] Schmid GP, Stoner BP, Hawkes S, Broutet N. The need and plan for global elimination of congenital syphilis. Sex Transm Dis. 2007;34: S5–10. 10.1097/01.olq.0000261456.09797.1b 17592390

[6] Gloyd S, Chai S, Mercer MA. Antenatal syphilis in sub-Saharan Africa: missed opportunities for mortality reduction. 2001;16: 29–34. 11238427 10.1093/heapol/16.1.29

[7] Finelli L, Berman SM, Koumans EH, Levine WC. Congenital syphilis. Bull World Health Organ. 1998;76 Suppl 2: 126–128. Available: http://www.ncbi.nlm.nih.gov/pubmed/10063689 10063689 PMC2305700

[8] Lawn JE, Blencowe H, Pattinson R, Cousens S, Kumar R, Ibiebele I, et al Stillbirths: Where? When? Why? How to make the data count? Lancet. 2011;377: 1448–1463. 10.1016/S0140-6736(10)62187-3 21496911

[9] Mwapasa V, Rogerson SJ, Kwiek JJ, Wilson PE, Milner D, Molyneux ME, et al Maternal syphilis infection is associated with increased risk of mother-to-child transmission of HIV in Malawi. AIDS. 2006;20: 1869–1877. 10.1097/01.aids.0000244206.41500.27 16954728

[10] Wilkinson D, Sach M, Connolly C. Epidemiology of syphilis in pregnancy in rural South Africa: opportunities for control. Trop Med Int Heal TM IH. 1997;2: 57–62. Available: http://www.ncbi.nlm.nih.gov/pubmed/9018303 9018303 10.1046/j.1365-3156.1997.d01-127.x

[11] Potter D, Goldenberg RL, Read JS, Wang J, Hoffman IF, Saathoff E, et al Correlates of syphilis seroreactivity among pregnant women: the HIVNET 024 Trial in Malawi, Tanzania, and Zambia. Sex Transm Dis. 2006;33: 604–609. 10.1097/01.olq.0000216029.00424.ae 16601659 PMC2743105

[12] Chico R, Mayaud P, Ariti C, Mabey D, Ronsmans C, Chandramohan D. Prevalence of malaria and sexually transmitted and reproductive tract infections in pregnancy in sub-saharan africa: A systematic review. JAMA. 2012;307: 2079–2086. 10.1001/jama.2012.3428 22665107

[13] World Health Organization Provision of Effective Antenatal Care: Integrated Management of Pregnancy and Childbirth (IMPAC). Geneva, Switzerland: Department of Making Pregnancy Safer (MPS) World Health Organization (WHO); 2002.

[14] Hawkes S, Matin N, Broutet N, Low N. Effectiveness of interventions to improve screening for syphilis in pregnancy: a systematic review and meta-analysis. Lancet Infect Dis. 2011;11: 684–691. 10.1016/S1473-3099(11)70104-9 21683653

[15] Watson-Jones D, Gumodoka B, Weiss H, Changalucha J, Todd J, Mugeye K, et al Syphilis in pregnancy in Tanzania. II. The effectiveness of antenatal syphilis screening and single-dose benzathine penicillin treatment for the prevention of adverse pregnancy outcomes. J Infect Dis. 2002;186: 948–957. 10.1086/342951 12232835

[16] Deperthes BD, Meheus A, O’Reilly K, Broutet N. Maternal and congenital syphilis programmes: case studies in Bolivia, Kenya and South Africa. Bull World Health Organ. 2004;82: 410–416. Available: http://www.ncbi.nlm.nih.gov/pmc/articles/PMC2622863/ 15356932 PMC2622863

[17] Peeling RW, Mabey D, Fitzgerald DW, Watson-Jones D. Avoiding HIV and dying of syphilis. Lancet. 2004;364: 1561–1563. 10.1016/S0140-6736(04)17327-3 15519615

[18] Central Statistical Office (CSO) M of H (MOH), Tropical Disease Research Centre (TDRC) U of Z, Inc. MI. Zambia Demographic and Health Survey 2007. [Internet]. Calverton, Maryland, USA: CSO and Macro International Inc.; 2009. Available: http://countryoffice.unfpa.org/zambia/drive/2007_zdhs_final_report.pdf

[19] Blandford JM, Gift TL, Vasaikar S, Mwesigwa-Kayongo D, Dlali P, Bronzan RN. Cost-effectiveness of on-site antenatal screening to prevent congenital syphilis in rural eastern Cape Province, Republic of South Africa. Sex Transm Dis. 2007;34: S61–66. 10.1097/01.olq.0000258314.20752.5f 17308502

[20] Terris-Prestholt F, Watson-Jones D, Mugeye K, Kumaranayake L, Ndeki L, Weiss H, et al Is antenatal syphilis screening still cost effective in sub-Saharan Africa. Sex Transm Infect. 2003;79: 375–381. 10.1136/sti.79.5.375 14573832 PMC1744759

[21] Mabey D, Peeling RW, Ballard R, Benzaken AS, Galban E, Changalucha J, et al Prospective, multi-centre clinic-based evaluation of four rapid diagnostic tests for syphilis. Sex Transm Infect. 2006;82: v13–v16. 10.1136/sti.2006.022467 17215274 PMC2563907

[22] Bates I, Maitland K. Are laboratory services coming of age in sub-Saharan Africa? Clin Infect Dis an Off Publ Infect Dis Soc Am. 2006;42: 383–384. 10.1086/499368 16392085

[23] Herring A, Ballard R, Mabey D, Peeling RW, Initiative WSTDD. Evaluation of rapid diagnostic tests: syphilis. Nat Rev Microbiol. 2006;4: S33–40. 10.1038/nrmicro1563 17366685

[24] Loubiere S, Moatti J-P. Economic evaluation of point-of-care diagnostic technologies for infectious diseases. Clin Microbiol Infect Off Publ Eur Soc Clin Microbiol Infect Dis. 2010;16: 1070–1076. 10.1111/j.1469-0691.2010.03280.x 20670289

[25] Palamountain KM, Baker J, Cowan EP, Essajee S, Mazzola LT, Metzler M, et al Perspectives on Introduction and Implementation of New Point-of-Care Diagnostic Tests. J Infect Dis. 2012;205: S181–S190. 10.1093/infdis/jis203 22402038 PMC3334510

[26] Shott JP, Galiwango RM, Reynolds SJ. A Quality Management Approach to Implementing Point-of-Care Technologies for HIV Diagnosis and Monitoring in Sub-Saharan Africa. J Trop Med. 2012;2012: 651927 10.1155/2012/651927 22287974 PMC3263631

[27] Chandler CIR, Mangham L, Njei AN, Achonduh O, Mbacham WF, Wiseman V. “As a clinician, you are not managing lab results, you are managing the patient”: how the enactment of malaria at health facilities in Cameroon compares with new WHO guidelines for the use of malaria tests. Soc Sci Med. 2012;74: 1528–1535. 10.1016/j.socscimed.2012.01.025 22430000

[28] Chandler CIR, Whitty CJM, Ansah EK. How can malaria rapid diagnostic tests achieve their potential? A qualitative study of a trial at health facilities in Ghana. Malar J. 2010;9: 95 10.1186/1475-2875-9-95 20398262 PMC2859355

[29] Reynolds J, Wood M, Mikhail A, Ahmad T, Karimullah K, Motahed M, et al Malaria “diagnosis” and diagnostics in Afghanistan. Qual Health Res. 2013;23: 579–591. 10.1177/1049732312470761 23275460

[30] Yao K, Wafula W, Bile EC, Cheignsong R, Howard S, Demby A, et al Ensuring the quality of HIV rapid testing in resource-poor countries using a systematic approach to training. Am J Clin Pathol. 2010;134: 568–572. 10.1309/AJCPOPXR8MNTZ5PY 20855637

[31] Parekh BS, Kalou MB, Alemnji G, Ou C-Y, Gershy-Damet G-M, Nkengasong JN. Scaling up HIV rapid testing in developing countries: comprehensive approach for implementing quality assurance. Am J Clin Pathol. 2010;134: 573–584. 10.1309/AJCPTDIMFR00IKYX 20855638

[32] Peeling RW, Holmes KK, Mabey D, Ronald A. Rapid tests for sexually transmitted infections (STIs): the way forward. Sex Transm Infect. 2006;82 Suppl 5: v1–6. 10.1136/sti.2006.024265 17151023 PMC2563912

[33] Gloyd S, Montoya P, Floriano F, Chadreque MC, Pfeiffer J, Gimbel-Sherr K. Scaling up antenatal syphilis screening in Mozambique: transforming policy to action. Sex Transm Dis. 2007;34: S31–36. 10.1097/01.olq.0000264586.49616.72 17592388

[34] Mabey DC, Sollis KA, Kelly HA, Benzaken AS, Bitarakwate E, Changalucha J, et al Point-of-care tests to strengthen health systems and save newborn lives: the case of syphilis. PLoS Med. 2012;9: e1001233 10.1371/journal.pmed.1001233 22719229 PMC3373627

[35] Sweeney S, Mosha JF, Terris-Prestholt F, Sollis KA, Kelly H, Changalucha J, et al The costs of accessible quality assured syphilis diagnostics: informing quality systems for rapid syphilis tests in a Tanzanian setting. Health Policy Plan. 2013; 10.1093/heapol/czt049 23894075

[36] Ansah EK, Reynolds J, Akanpigbiam S, Whitty CJM, Chandler CIR. “Even if the test result is negative, they should be able to tell us what is wrong with us”: a qualitative study of patient expectations of rapid diagnostic tests for malaria. Malar J. Malaria Journal; 2013;12: 258 10.1186/1475-2875-12-258 PMC3723648 23876112

[37] Asiimwe C, Kyabayinze DJ, Kyalisiima Z, Nabakooza J, Bajabaite M, Counihan H, et al Early experiences on the feasibility, acceptability, and use of malaria rapid diagnostic tests at peripheral health centres in Uganda-insights into some barriers and facilitators. Implement Sci IS. 2012;7: 5 10.1186/1748-5908-7-5 22269037 PMC3398266

[38] WHO. WHO Country Statistics Zambia. In: Country Statistics [Internet]. 2014. Available: http://www.who.int/countries/zmb/en/

[39] Observatory GH. Zambia: Health Profile. In: Zambia Country Health Profile [Internet]. 2012. Available: http://www.who.int/gho/countries/zmb.pdf?ua=1

[40] Ferrinho P, Siziya S, Goma F, Dussault G. The human resource for health situation in Zambia: deficit and maldistribution Hum Resour Health. 2011;9: 30 10.1186/1478-4491-9-30 22182366 PMC3283521

[41] Strasser S, Bitarakwate E, Gill M, Hoffman HJ, Musana O, Phiri A, et al Introduction of rapid syphilis testing within prevention of mother-to-child transmission of HIV programs in Uganda and Zambia: a field acceptability and feasibility study. J Acquir Immune Defic Syndr. 2012;61: e40–46. 10.1097/QAI.0b013e318267bc94 22820810

[42] Zambian MoH. National programme for the prevention and control of sexually transmitted infection: guidelines for use of rapid syphilis tests in Zambia. Lusaka; 2011.

[43] Shelley KD, Ansbro ÉM, Tshaka Ncube A, Sweeney S, Fleischer C, Tembo Mumba G, et al Scaling down to scale up: a health economic analysis of integrating point-of-care syphilis testing into antenatal care in Zambia during pilot and national rollout implementation. Rev. 2015; 10.1371/journal.pone.0125675 PMC4430530 25970443

[44] Chuttur MY. Overview of the Technology Acceptance Model: Origins, Developments and Future Directions [Internet]. Indiana University, USA, Sprouts: Working Papers on Information Systems; 2009. Available: http://sprouts.aisnet.org/9-37

[45] Jeng J. What Is Usability in the Context of the Digital Library and How Can It Be Measured. Inf Technol Libr. 2013;24: 47–56. 10.6017/ital.v24i2.3365

[46] King N. Template Analysis [Internet]. School of Human Sciences, University of Huddersfield; 2012. Available: http://hhs.hud.ac.uk/w2/research/template_analysis/index.htm

[47] Broome C, Adams A. What gets missed when deploying new technologies in A&E? Med Inform Internet Med. 2005;30: 83–87. 10.1080/14639230500298750 16338796

[48] Jafari Y, Johri M, Joseph L, Vadnais C, Pant Pai N. Poor Reporting of Outcomes Beyond Accuracy in Point-of-Care Tests for Syphilis: A Call for a Framework. AIDS Res Treat. 2014;2014: 465932 10.1155/2014/465932 24795821 PMC3985157

[49] Schito M, Peter TF, Cavanaugh S, Piatek AS, Young GJ, Alexander H, et al Opportunities and challenges for cost-efficient implementation of new point-of-care diagnostics for HIV and tuberculosis. J Infect Dis. 2012;205 Suppl: S169–180. 10.1093/infdis/jis044 22457286 PMC3334507

[50] Rafi M. Evaluating training cascade: a methodology and case study. Educ Res Rev. 2010;5: 64–77. Available: http://academicjournals.org/article/article1379603319_Rafi.pdf

[51] Kyabayinze DJ, Asiimwe C, Nakanjako D, Nabakooza J, Bajabaite M, Strachan C, et al Programme level implementation of malaria rapid diagnostic tests (RDTs) use: outcomes and cost of training health workers at lower level health care facilities in Uganda. BMC Public Health. 2012;12: 291 10.1186/1471-2458-12-291 22519958 PMC3433367

[52] Alliance GHW. Scaling Up, Saving Lives. Task Force for Scaling Up Education and Training for Health Workers [Internet]. World Health Orgnisation; 2008. Available: http://www.who.int/workforcealliance/documents/Global_HealthFINALREPORT.pdf?ua=1

[53] Pearce J, Mann MK, Jones C, van Buschbach S, Olff M, Bisson JI. The most effective way of delivering a train-the-trainers program: a systematic review. J Contin Educ Health Prof. 2012;32: 215–226. Available: http://www.ncbi.nlm.nih.gov/pubmed/23173243 23173243 10.1002/chp.21148

[54] Ridde V. Per diems undermine health interventions, systems and research in Africa: burying our heads in the sand. Trop Med Int Heal TM IH. 2010; 10.1111/j.1365-3156.2010.02607.x 28639744

[55] Vian T, Miller C, Themba Z, Bukuluki P. Perceptions of per diems in the health sector: evidence and implications. Health Policy Plan. 2013;28: 237–246. 10.1093/heapol/czs056 22684639 PMC3643111

[56] García SG, Tinajeros F, Revollo R, Yam EA, Richmond K, Díaz-Olavarrieta C, et al Demonstrating public health at work: a demonstration project of congenital syphilis prevention efforts in Bolivia. Sex Transm Dis. 2007;34: S37–41. 10.1097/01.olq.0000251236.48770.35 17179776

[57] Blattner K, Nixon G, Jaye C, Dovey S. Introducing point-of-care testing into a rural hospital setting: thematic analysis of interviews with providers. J Prim Health Care. 2010;2: 54–60. Available: http://www.ncbi.nlm.nih.gov/pubmed/20690403 20690403

[58] De Savigny D, Adam T. Systems thinking for health system strengthening [Internet]. Alliance for Health Policy and Systems Research, WHO; 2009 Available: http://whqlibdoc.who.int/publications/2009/9789241563895_eng.pdf

[59] Bristow CC, Adu-Sarkodie Y, Ondondo RO, Bukusi EA, Dagnra CA, Oo KY, et al Multisite Laboratory Evaluation of a Dual Human Immunodeficiency Virus (HIV)/Syphilis Point-of-Care Rapid Test for Simultaneous Detection of HIV and Syphilis Infection. Open Forum Infect Dis. 2014;1: ofu015 10.1093/ofid/ofu015 25734088 PMC4324189

[60] Chandler CIR, Chonya S, Boniface G, Juma K, Reyburn H, Whitty CJM. The importance of context in malaria diagnosis and treatment decisions—a quantitative analysis of observed clinical encounters in Tanzania. Trop Med Int Health. 2008;13: 1131–42. 10.1111/j.1365-3156.2008.02118.x 18631313

[61] Plate DK. Evaluation and Implementation of Rapid HIV Tests: The Experience in 11 African Countries. AIDS Res Hum Retroviruses. 2007;23: 1491–1498. 10.1089/aid.2007.0020 18160006

[62] Yamey G. Scaling Up Global Health Interventions: A Proposed Framework for Success. PLoS Med. 2011;8: e1001049 10.1371/journal.pmed.1001049 21738450 PMC3125181

