# Supplementary material for: Introduction of Syphilis Point-of-Care Tests, from Pilot Study to National Programme Implementation in Zambia: A Qualitative Study of Healthcare Workers’ Perspectives on Testing, Training and Quality Assurance
Source: PLoS One. 2015 Jun 1;10(6):e0127728. doi: 10.1371/journal.pone.0127728 (PMC4452097; doi:10.1371/journal.pone.0127728)
Supplement: S1 Appendix — (PDF) [file pone.0127728.s001.pdf]

Zambian RST Health Care Worker Questionnaire

Questionnaire: Topic Guide for Qualitative Section

In this section I will ask you a set of open-ended questions. There are no right or wrong answers. I am interested in your own opinions and experiences of using rapid syphilis tests in this facility.

A. Warm-up / Introduction

Can you tell me about a typical day working in this health facility as a (nurse)?

(PROMPT: Can you tell me about a typical day when you use rapid syphilis tests? Do you them in this clinic or in the laboratory; which health workers perform the tests.
What has been your experience so far in using the rapid syphilis tests in this health centre?

B. Perception of Tests / Culture

1. How has your practice been affected by the introduction of the new rapid syphilis tests?

(PROMPT: How has your working day as a (nurse) been affected by the introduction of the new rapid syphilis test? How has it affected your workload? Do you think you are more or less busy since the test was introduced?)

2. Have you had any problems using the rapid syphilis test? **Circle: Yes No**

(PROMPT: Can you tell me about the most difficult time you had when using the rapid syphilis test? can you tell me about the best or any particularly good time you had when using RSTs)

3. Since the rapid syphilis test was introduced here, do you think there have been any benefits to health clinic staff? **Circle: Yes / No**

(PROMPT: If yes, can you tell me about the benefits? How have they helped HCWs? What aspects of your work have become easier? Which staff members have been affected?)

4. Have there been any problems/ disadvantages for health clinic staff? **Circle: Yes / No**

(PROMPT: If yes, can you tell me about them? What are the drawbacks for staff? What aspects of you work have become more difficult? Which staff members have been affected in this way? How do they deal with difficulties?)

C. Knowledge / Training

5. Did you receive training in the use of Rapid Syphilis Tests before using them? **Yes / No**

(PROMPT: Please describe the training received: by whom, where, how long? What your experience of this training or orientation; how did you find it? Were there any good or bad things about it? How did you think orientation differs from training? Is there anything you would change in the way you were taught to use RSTs? If you were designing a training programme for HCWs in another district how would you recommend it should be done?)

6. Can you describe for me exactly how you carry out a rapid syphilis test?

(PROMPT: What are the steps you follow? Do you follow any guideline or look at any written material while you perform the test? Do you do it from memory? Do you use job aids or posters on the wall? Is there a guideline available in the facility; have you seen it or read it?)

D. Social Environment

7. How have patients been affected by the introduction of the new rapid syphilis tests?

(PROMPT: What have been the benefits or advantages for patients? Have there been any difficulties from a patient point of view? How have patients responded to the introduction of the new Rapid Syphilis Tests?)
8. What is your experience of partner notification for women who test syphilis positive using the RST?

(PROMPT: How is it done in this facility? have there been any difficulties with partner notification. Can you tell me about the most difficult time you had with partner notification. How did you deal with that?)

E. Supervision
9. Since rapid syphilis tests were introduced in this centre, have you experienced any supervisory visits? **Circle: Yes / No**

Can you tell me about your experience of supervisory visits? (PROMPT: Have you experienced supervisory visits for any other services you provide in the clinic? Can you tell me about them? Who does them, how often? Are there any good or bad aspects?)

F. Quality System
10. Have you taken part in any Quality Assurance activities since the introduction of rapid syphilis tests into this health centre? **Circle: Yes / No**

(PROMPT: What does Quality Assurance mean to you? Have you experienced quality assurance for any other service that takes place in this facility? Can you tell me exactly how Quality Assurance (QA) activities are carried out...how is it done, by whom, how often? How are the results acted on? Can you think of any benefits of the Quality Assurance part of the RST programme? Can you tell me about them? Can you think of any problems with the Quality Assurance part of the RST programme? Can you tell me about them?

11. Have you taken part of any Quality Control activities since RSTs were introduced into this health centre? **Circle: Yes / No**

(PROMPT: What does Quality Control mean to you? Can you tell me exactly how the Quality Control procedure is done in this health centre how is it done… by whom, how often? Is it done for other types of tests that you use? Can you think of any good things about QC testing? Can you tell me about them? Can you think of any problems with QC Testing? Can you tell me about them?

G. Future of RST Programme

12. Can you think of any barriers to carrying out RST testing at this facility?

(PROMPT: Is there anything missing? Is there anything that gets in the way of carrying out RSTs? Do you think testing should continue at this facility?
13. Can you think of anything that would facilitate or support the staff in this facility in carrying out rapid syphilis tests? Is there anything the MOH can do to help it continue at this facility?

14. Do you have any other message for the Ministry of Health about the RST Programme before it is rolled out to other districts in Zambia?

END OF QUALITATIVE SECTION. GO TO QUANTITATIVE SURVEY.

PART B **Quantitative Survey Part A: Demographic Data and Experience with Rapid Syphilis Testing**

No.	Question	Answer		Variable
1.1	Date of interview	____ /___/12		Date
1.2	Interviewer initials	|__||__|		IntID
1.3	Survey ID number (unique)	|__||__|		SurvID
1.4	Name of health centre	______________________		centre
1.5	District		1<br>2	District
1.6	What is your occupation in this health centre?	HIV counsellor	0	HcwRole
		Midwife	1	
		Nurse	2	
		Lab Technician	3	
		Medical Officer	4	
		Clinical Officer	5	
		Counsellor	6	
		Other, Specify: __________	7	
		Don't Know	8	
		Refuse	9	
1.7	How many years of professional experience do you have in this field?	Years: |__||__| Months: |__||__|		HcwYr
1.8	Since the introduction of rapid syphilis testing at your health centre, have you personally administered any rapid syphilis tests?<br>**Circle only ONE**<br>**If No, skip to Q 2.1**	Yes	1	RstUse
		No	2	
		Don't Know	8	
		Refuse	9	
1.9	Approximately how many patients have you tested with the rapid syphilis test during the last one month?<br>**Circle only ONE**	1-10 patients	1	RstQty
		11-20 patients	2	
		21-30 patients	3	
		More than 30 patients	4	
		None	5	
		Don't Know	8	
		Refuse	9	

Quantitative Survey Part B: Integrating Rapid Syphilis Testing with Clinical Workflow

No.	Question	Answer		Variable
2.1	What method do you use for collecting blood samples for rapid HIV and syphilis testing?<br>**Read all choices; Circle only ONE**	Finger prick	1	BloodCol
		Venous blood draw	2	
		Other, Specify: _____________	3	OthBlCol
		Don't Know	8	
		Refuse	9	
2.2	On a typical day at your facility, are rapid syphilis and HIV tests run by the same health care worker or different health care workers?<br>**Read all choices: circle only ONE**	Rapid syphilis & HIV tests are run by different health care workers	1	TestPr
		Rapid syphilis & HIV tests are run by the same health care worker	2	
		Don't Know	8	
		Refuse	9	
2.3	*QUALITATIVE:* Please provide additional details on testing process for HIV and syphilis testing in the space below (i.e. batched tests, run side by side etc):			OthTstPr
2.4	Do you think rapid syphilis testing has been successfully integrated with PMTCT services, including HIV counselling and testing, at this clinic?<br>**Circle only ONE**	Yes	1	Combine
		No	2	
		No Opinion	3	
		Don't Know	8	
		Refuse	9	
2.5	Do you think adding rapid syphilis testing onto HIV testing has changed patient waiting time at<br>**Read all choices; Circle only ONE**<br>**If 1, go to Q. 2.6**<br>**If 2, go to Q. 2.7**	Patients will have to wait <u>more</u> time	1	Waiting
		Patients will have to wait <u>less</u> time	2	
		No change in patient wait time	3	
		Don't Know	8	
		Refuse	9	
2.6	How much additional time do you think women have to wait to get syphilis test results?<br>**Read all choices; Circle only ONE**	1 to 10 minutes extra time	1	AddTime
		11 to 20 minutes extra	2	
		21 to 40 minutes extra	3	
		Over 40 minutes extra	4	
		N/A	5	
		Don't Know	8	
		Refuse	9	
2.7	The majority of the time, after positive syphilis test results are given, is treatment started on the same day?<br>**Read all choices; Circle only ONE**<br>**If yes, go to Q2.9**	Yes (Same day)	1	TxSame
		No (Different day)	2	
		Don't Know	8	
		Refuse	9	

No.	Question	Answer		Variable
2.8	*If No to Q 2.7:* What was the main reason treatment was not given on the same day? **Read all choices; Circle only ONE**	Penicillin stock out	1	ResNoTx
		Staff not available to give treatment	2	
		Client allergic to penicillin and no alternative available	3	
		Client did not consent	4	
		Other: Specify______________	5	OthResNT
		Don't Know	8	
		Refuse	9	
2.9	How often were there not enough test kits to carry out the rapid syphilis testing in the past one month? **Read all choices; Circle only ONE**	Never	1	RstStock
		About once a week	2	
		About twice a month	3	
		About once a month	4	
		Whole month	5	
		Don't Know	8	
		Refuse	9	
2.10	How often was there not enough penicillin to treat women or their partners who tested positive for syphilis in the past one month? **Read all choices; Circle only ONE**	Never	1	BPStock
		About once a week	2	
		About twice a month	3	
		About once a month	4	
		Don't Know	8	
		Refuse	9	
2.11	Was any other equipment not available to perform the RST during the past month? **Read all choices; Circle any relevant**	Gloves	1	Equip
		Sharps box	2	
		Lancets for finger prick	3	
		Clock / Timer	4	
		Other__________________	3	Equip Other
		Don't Know	4	
		Refuse	5	
2.12	Before the introduction of the rapid syphilis test, was routine syphilis testing being done at this facility? **Read all choices; Circle only ONE** **If no, go to Q3.1**	Yes	1	Routine
		No	2	
		Don't Know	8	
		Refuse	9	
2.13	If Yes, by what method? **Read all choices; Circle only ONE**	RPR	1	PrevTest
		VDRL	2	
		Rapid Syphilis Tests	3	
		N/A	4	
		Don't Know	8	
		Refuse	9	

Quantitative Survey Part C: Training and JobAids for Performing Rapid Syphilis Test

No.	Question	Answer		Variable
	**Skip to PART E if HCW never performed the RST**			
3.1	**Have you received training in rapid syphilis testing?**<br>**Read all choices, Circle only ONE**<br>**If yes, go to Q. 3.2**<br>**If no, go to Q. 3.3**	Yes<br>No<br>Don't Know<br>Refuse	1<br>2<br>8<br>9	Training
3.2	**What type of training did you receive in using the rapid syphilis test?**<br>**Circle Yes/No for each option.**			
	Training event with EGPAF/ MOH	Yes<br>No<br>DK	1<br>2<br>8	TrnType1
	On the job training by a colleague (practice, supervision & correction)	Yes<br>No<br>DK	1<br>2<br>8	TrnType2
	Follow-up training session	Yes<br>No<br>DK	1<br>2<br>8	TrnType3
	Other: Specify_______________________	Yes<br>No<br>DK	1<br>2<br>8	OthTrain
3.3	**What job aid(s) have you used when performing the RST?**<br>**Circle Yes/No for each option.**			
	Standard Operating Procedures (SOPs)	Yes<br>No<br>DK	1<br>2<br>8	JobAid1
	Flip Chart	Yes<br>No<br>DK	1<br>2<br>8	JobAid2
	Manufacturer's product insert	Yes<br>No<br>DK	1<br>2<br>8	JobAid3
	Posters	Yes<br>No<br>DK	1<br>2<br>8	JobAid4
	Other: Specify ____________________	Yes<br>No<br>DK	1<br>2<br>8	OthAid5
3.4	**Is this job aid visible at your work station?**<br>**Read all options; Circle only ONE**	Yes<br>No<br>DK	1<br>2<br>8	VisJAid

Quantitative Survey Part D: Learnability of Rapid Syphilis Testing

No.	Question	Answer		Variable
	**The interviewer should ask the HCW to rate the statements using the following scale: very difficult, quite difficult, ok, quite easy, or very easy.**			
4.1	Overall, performing a rapid syphilis test is:<br>**Read all choices; Circle only ONE**	Very Difficult<br>Quite Difficult<br>OK<br>Quite Easy<br>Very Easy<br>Don't Know<br>Refuse	1<br>2<br>3<br>4<br>5<br>8<br>9	DiffPre
	**Interviewer should not ask the HCW this question**<br>*See question 2.1 for answer*<br>If finger prick , skip to **Q4.2**<br>If blood draw skip to **Q4.3**	Finger prick<br>Venous blood draw<br>Other, Specify: ____________<br>Don't Know<br>Refuse	1<br>2<br>3<br>8<br>9	BloodCol
4.2	Collecting the blood sample by finger prick for the rapid syphilis test is:<br>**Read all choices; Circle only ONE**	Very Difficult<br>Quite Difficult<br>OK<br>Quite Easy<br>Very Easy<br>N/A – not involved in this aspect<br>Don't Know<br>Refuse	1<br>2<br>3<br>4<br>5<br>6<br>8<br>9	DiffLanc
4.3	Collecting the blood sample by venipuncture for the rapid syphilis test is:<br>**Read all choices; Circle only ONE**	Very Difficult<br>Quite Difficult<br>OK<br>Quite Easy<br>Very Easy<br>N/A – not involved in this aspect<br>Don't Know<br>Refuse	1<br>2<br>3<br>4<br>5<br>6<br>8<br>9	DiffVeni
4.4	Using the micro-pipette for the blood sample is:<br>**Read all choices; Circle only ONE**	Very Difficult<br>Quite Difficult<br>OK<br>Quite Easy<br>Very Easy<br>N/A – not involved in this aspect<br>Don't Know<br>Refuse	1<br>2<br>3<br>4<br>5<br>6<br>8<br>9	DiffPpt

No.	Question	Answer		Variable
4.5	Adding the correct amount of blood sample onto the absorbent pad of the RST is:<br>**Read all choices; Circle only ONE**	Very Difficult<br>Quite Difficult<br>OK<br>Quite Easy<br>Very Easy<br>N/A – not involved in this aspect<br>Don't Know<br>Refuse	1<br>2<br>3<br>4<br>5<br>6<br>8<br>9	DiffAmt
4.6	Adding the correct amount of buffer solution to the specimen well of the RST is:<br>**Read all choices; Circle only ONE**	Very Difficult<br>Quite Difficult<br>OK<br>Quite Easy<br>Very Easy<br>N/A – not involved in this aspect<br>Don't Know<br>Refuse	1<br>2<br>3<br>4<br>5<br>6<br>8<br>9	DiffBuff
4.7	Accurately timing the rapid syphilis test before reading and recording the results is:<br>**Read all choices; Circle only ONE**	Very Difficult<br>Quite Difficult<br>OK<br>Quite Easy<br>Very Easy<br>N/A – not involved in this aspect<br>Don't Know<br>Refuse	1<br>2<br>3<br>4<br>5<br>6<br>8<br>9	DiffTime
4.8	Correctly reading and interpreting the rapid syphilis test results is:<br>**Read all choices; Circle only ONE**	Very Difficult<br>Quite Difficult<br>OK<br>Quite Easy<br>Very Easy<br>N/A – not involved in this aspect<br>Don't Know<br>Refuse	1<br>2<br>3<br>4<br>5<br>6<br>8<br>9	DiffRslt
4.9	Interpreting weak positive test results?<br>**Read all choices; Circle only ONE**	Very Difficult<br>Quite Difficult<br>OK<br>Quite Easy<br>Very Easy<br>N/A – not involved in this aspect<br>Don't Know<br>Refuse	1<br>2<br>3<br>4<br>5<br>6<br>8<br>9	DiffWeak

Quantitative Survey Part E: Acceptability of Rapid Syphilis Testing, QA and QC Activites

No.	Question	Answer		Variable
5.1	In your opinion, how do new patients feel about the rapid syphilis test?<br>**Read all choices: Circle only ONE**	Very negative	1	LikeTest
		Quite negative	2	
		Ok	3	
		Quite positive	4	
		Very positive	5	
		Don't Know	8	
		Refuse	9	
5.2	Do you think routine rapid syphilis testing should continue in your health facility?<br>**Circle only ONE**<br>If yes, go to Q 5.3<br>If no, go to Q 5.4	Yes	1	RSTSupp
		No	2	
		Don't Know	8	
		Refuse	9	
5.3	What is the most important reason you support use of RSTs at this facility?<br>**Read all choices: Circle only ONE**	Low cost	1	WhyYes
		Does not add to workload	2	
		Results are relible	3	
		Test is easy to carry out	4	
		Other, specify: ______________	5	
		Don't Know	8	
		Refuse	9	
5.4	What is the most important reason you don't support use of RSTs at this facility?<br>**Read all choices: Circle only ONE**	High cost	1	WhyNo
		Adds to workload	2	
		Results are unreliable	3	
		Test is difficult to carry out	4	
		Other, specify: ______________	5	
		N/A	6	
		Don't Know	8	
		Refuse	9	
5.5	Do you think the Quality Assurance procedures should happen at this facility?<br>**Read all choices: Circle only ONE**<br>If yes, go to Q 5.6<br>If no, go to Q 5.7	Yes	1	QASupp
		No	2	
		Don't Know	8	
		Refuse	9	
5.6	What is the most important reason you support the Quality Assurance continuing at your facility?<br>**Read all choices: Circle only ONE**	Low cost	1	QAYes
		Does not add to workload	2	
		Gives health worker confidence	3	
		Identifies the need for more training	4	
		QA test is easy to carry out	5	
		Other, specify: ______________	6	
		Don't Know	8	
		Refuse	9	

No.	Question	Answer		Variable
5.7	What is the main reason you do not support Quality Assurance testing at your facility?<br>**Read all choices: Circle only ONE**	High cost	1	QANo
		Adds to workload	2	
		Reduces health worker confidence	3	
		Identifies the need for more training	4	
		QA test is difficult to carry out	5	
		Other, specify: ______________	6	
		N/A	7	
		Don't Know	8	
		Refuse	9	
5.8	Do you think that Quality Control procedures should happen at this facility?<br>**Read all choices: Circle only ONE**<br>If 1, go to Q5.9<br>If 2, go to Q5.10	Yes	1	QCSupp
		No	2	
		Don't Know	8	
		Refuse	9	
5.9	What is the main reason you support Quality Control procedures at your facility?<br>**Read all choices: Circle only ONE**	Low cost	1	QCYes
		Does not add to workload	2	
		Add to HCW confidence in the RST	3	
		Patients get accurate results	4	
		QC test is easy to carry out	5	
		Other, specify: ____________	6	
		Don't Know	8	
		Refuse	9	
5.10	What is the main reason you do not support Quality Control procedures at your facility?<br>**Read all choices: Circle only ONE**	High cost	1	QCNo
		Adds to workload	2	
		Reduces HCW confidence in RST	3	
		Identifies problems with the test kit and invalidates patient results	4	
		Test is complicated to carry out	5	
		Other, specify: ______________	6	
		N/A	7	
		Don't Know	8	
		Refuse	9	
